# Living in Rural and Urban Areas of New Caledonia: Impact on Food Consumption, Sleep Duration and Anthropometric Parameters Among Melanesian Adolescents

**DOI:** 10.3390/nu12072047

**Published:** 2020-07-10

**Authors:** Olivier Galy, Emilie Paufique, Akila Nedjar-Guerre, Fabrice Wacalie, Guillaume Wattelez, Pierre-Yves Le Roux, Solange Ponidja, Paul Zongo, Christophe Serra-Mallol, Margaret Allman-Farinelli, Stéphane Frayon

**Affiliations:** 1 Interdisciplinary Laboratory for Research in Education, EA 7483, University of New Caledonia, Avenue James Cook, 98800 Nouméa, New Caledonia; emilie.paufique@etudiant.unc.nc (E.P.); akila.nedjar-guerre@unc.nc (A.N.-G.); fabrice.wacalie@unc.nc (F.W.); guillaume.wattelez@unc.nc (G.W.); pierre-yves.le-roux@unc.nc (P.-Y.L.R.); solange.ponidja@unc.nc (S.P.); lopops1070@hotmail.fr (P.Z.); stephanefrayon@hotmail.com (S.F.); 2 CERTOP—University of Toulouse Jean Jaurès, 5 Allée Antonio Machado, 31058 Toulouse, France; christophe.serra-mallol@univ-tlse2.fr; 3 Charles Perkins Centre, The University of Sydney, Camperdown, NSW 2006, Australia; margaret.allman-farinelli@sydney.edu.au

**Keywords:** food habits, nutrition behaviour, ethnicity, lifestyle, adolescents, sustainable development, Pacific

## Abstract

Background: Food consumption, sleep duration and overweight were assessed in rural and urban Melanesian adolescents. Methods: A cross-sectional survey of 312 rural and 104 urban adolescents (11–16 years old) was conducted. Food intakes were assessed by a 26-item food frequency questionnaire and then categorised into the number of serves from each of the three recommended Pacific food groups (energy foods, protective foods, bodybuilding foods), with two additional categories for foods and drinks to be avoided i.e., processed foods and sugary drinks. Number of food serves were compared with the guidelines of 50% serves from energy foods, 35% serves from protective foods and 15% serves from bodybuilding foods. Sleep duration as hours per day was self-reported and body mass index (BMI) was calculated from measured weight and height. Results: Approximately 17.9% of rural and 26.9% of urban adolescents met the guidelines for energy foods; 61.5% rural and 69.2% urban met the serves for protective foods and 88.5% and 94.2% met the serves for bodybuilding foods. Less than 6.4% rural and 1.9% urban adolescents avoided processed foods but 61.5% rural and 56.7% urban avoided sugary beverages. Sleep duration for school days was below the international recommendations and did not significantly differ between rural and urban groups: respectively, 8.16 ± 1.10 and 8.31 ± 1.29 h. Overweight/obesity percentage was 38.1% for rural and 31.7% for urban adolescents. Conclusions: Although traditional foods, including protective food, are still part of the adolescents’ diet, low consumption of the energy food group and high consumption of processed food occurs regardless of location. As poor eating habits and insufficient sleep may contribute to overweight/obesity, educational nutrition programs should target these lifestyle variables.

## 1. Introduction

Pacific Island Countries and Territories (PICTs) have been undergoing a brutal socioeconomic transition over the past 70 years. Pacific cultures have been exposed to a military presence during and after World War II [1], the development of centralised political rule, monetisation of economic systems and increased trade globalisation. Clearly, a lifestyle transition has been underway, and a diet once based on fresh seafood, vegetables and tubers has shifted to include canned meat or fish, oil, sugar, rice and processed foods [2]. At the same time, daily activity, which was once based on fishing and agriculture, has shifted to more sedentary activities that have had a major impact on health [3]. More recently, the mechanisation and digitisation of environments have also influenced daily behaviour and activity, including physical activity and sleep duration. Indeed, when sleep is less than optimal, energy expenditure is affected: sleep-deprived individuals are prone to feel sleepy and tired in the daytime, thus preferring sedentary activities to physical activities, which then lowers the energy expenditure [4]. Sleep deprivation negatively impacts metabolism, with rises in the hunger hormone ghrelin and increases in energy intake, particularly poor-nutrient energy-dense foods, as reported in Western populations [5,6,7,8]. These combined lifestyle variables are the root cause (but not unique) of noncommunicable diseases, and the young Pacific population is extremely exposed. The prevalence of overweight and obesity is very high in New Caledonian adolescents (from 36% to 43%, depending on age and the reference used to assess overweight) [9,10,11], and this is particularly the case for Melanesians. Indeed, a recent study showed that the prevalence of overweight/obesity was higher in 11 to 16-year-old Melanesian and Polynesian adolescents than in Caucasian adolescents, respectively, 38.2%, 30.4% and 21.3% [12].

In New Caledonia, where per capita income is much higher than in other PICTs [13], small-scale family farming is the predominant form of the agricultural system, particularly in the Loyalty Islands and Northern Province, inhabited mostly by the Melanesian people. In Melanesian culture, family farming remains prevalent [14], although sometimes household members leave the tribe to seek work in towns. Agricultural activities, hunting and fishing remain strong, despite the proliferation of development hubs, rising education levels and improved living conditions [15,16]. Nevertheless, young people continue to be exposed to new food environments and have thus enlarged their food choices and diversified their eating habits in both positive and negative ways [17]. Emergent food environments in low-to-middle-income countries have created conditions that facilitate the choice of lower-cost, less-healthy, more energy-dense foods, which may lead to overweight and obesity as access to healthy foods diminishes [18]. The Pacific Guidelines for Healthy Living provide advice about diet, physical activity, smoking and alcohol. These guidelines outline the proportions of foods to be consumed from three ‘healthy’ food groups (energy, protective and bodybuilding) and indicate the foods that should be limited. Water is the beverage of choice and sugar-sweetened beverages (SSBs) should be avoided [19]. Comparing food intakes with these guidelines can yield valuable insight into the food environments that these Pacific communities are experiencing [19]. In New Caledonia, another way to gain insight into the effects of the ongoing lifestyle transition might be to determine the proportion of ‘healthy foods’ versus ‘limited foods’ consumed by adolescents living in rural versus urban areas. In this context, ‘healthy food’ consumption can be defined as eating a variety of fresh local foods from the three food groups in the appropriate amounts each day (energy: 50% of food, protective: 35% and bodybuilding: 15%) and limiting food and beverages high in salt, sugar and fat. This means that imported processed food/drinks from the food industry should only be eaten in small amounts. Recent studies have demonstrated that the lifestyles of New Caledonian adolescents have undergone striking changes, characterised by a preference for highly processed drinks like SSBs [20], breakfast skipping [21] and relatively low physical activity [22]. These changes may have contributed to the prevalence of overweight and obesity in Melanesian adolescents, especially those living in rural areas, although no study has yet investigated this hypothesis. Yet Melanesian girls from rural areas were found to be less physically active than their urban counterparts [10], and this may result in the higher prevalence of overweight and obesity as previously observed for body fat mass with 27.5% and 23.9% in rural and urban adolescents of similar age [10]. In addition, sleep behaviour is widely associated with overweight and obesity, and several associated factors, like the influence of media at home in the evening and school transport in isolated areas, impact sleep duration [23]. We therefore hypothesised that food consumption, with the respective contributions of ‘healthy food’ and ‘limited food’, and sleep duration would differ according to the living environment (i.e., urban and rural) of the Melanesian adolescents and have an impact on anthropometric parameters.

This study aimed to assess food consumption, sleep duration and anthropometric parameters of Melanesian adolescents living in rural and urban areas in New Caledonia to provide baseline measures as the Pacific region undergoes transition. 

## 2. Materials and Methods

### 2.1. Data Collection and Participants

This research is part of a community-based food culture project underway in New Caledonia and its provinces: Northern Province, Southern Province and Loyalty Islands. All differ substantially in terms of ethnic distribution, socioeconomic status and urbanisation. The ethnic groups are as follows: Melanesian: 39.0%, European: 34.4%, Polynesian: 10.0%, Asian: 2.7% and other groups 14.1% [13]. The Melanesian community is distributed as follows: 77.0% live in rural areas and 23.0% in urban areas [13]. Forty percent of the public schools are in rural areas (*n* = 13) and 60% in urban areas (*n* = 20) [13]. The criteria for selecting the schools for this study were (1) location (rural and urban), (2) sufficient school size (*n* > 200) and (3) the agreement of the school’s principal. Five schools were eligible in Southern Province (urban area), two in Northern Province (one on each coast) and only one in Loyalty Islands (Lifou Island). Participating classes were then randomly drawn from within these eligible schools. The school and participant selection processes are more fully described elsewhere [24].

We gathered data from July 2018 to April 2019 from 1060 adolescents from the community-based food culture project, 11 to 16 years old from several ethnic community. In the current study, only Melanesian adolescents were considered, providing a final sample of 416 Melanesian adolescents representing 39.2% of the total sample and reflecting the percentage of Melanesians in the New Caledonian population [13].

We obtained informed written consent from all parents before their children entered the study. The research met the legal requirements and the Declaration of Helsinki, and the protocol was approved by the Ethics Committee of the University of New Caledonia: CCE 2018-06 001.

### 2.2. Measures

#### 2.2.1. Anthropometric Parameters

A trained staff collected the anthropometric data in the school nurse’s office. A portable stadiometer (Leicester Tanita HR 001, Tanita Corporation, Tokyo, Japan) measured height to the nearest 0.1 cm. Weight was assessed to the nearest 0.1 kg using a scale (Tanita HA 503, Tanita Corporation, Tokyo, Japan), with the adolescents wearing light clothing. From these measurements, body mass index (BMI) was calculated as follows BMI = weight [kg]/([height [m])^2^. 

We used the International Obesity Task Force (IOTF) criteria for children to define the adolescents as thin (underweight), normal weight, overweight or obese. The IOTF criteria provide BMI cut-offs for weight status based on BMI values according to age and sex [25].

#### 2.2.2. Sociodemographic Characteristics

The adolescents used an anonymous survey tool to report ethnicity, and the ethnic groups were categorised following the recommendations from the report on New Caledonia [26] by the Institut National de la Santé Et de la Recherche Médicale (INSERM; National Institute of Health and Medical Research). Three SES categories were determined based on the National Statistics Socio-Economic Classification [27]: managerial and professional occupations (high), intermediate occupations (medium), and routine and manual occupations (low). We referred to the latest census in New Caledonia [13] and a European standard to determine the degrees of urbanisation [28]: Noumea and its suburbs were classified as urban and the other areas were classified as rural.

#### 2.2.3. Food Frequency Questionnaire (FFQ)

The short FFQ was adapted from the validated version of the FFQ for Aboriginal and Torres Strait Islanders by Gwynn et al. [29], in the absence of a validated FFQ for New Caledonia. Minor modifications were made by the research team to include foods identified as important in the diet of Melanesian adolescents [19] (Table 1). For example, tubers such as cassava, yams and taro are consumed rather than white potatoes, and a common snack food is reconstituted noodle soup, e.g., Maggi noodles. The FFQ contains 26 questions on food and beverage intake with additional questions on the purchase of food on the journey to and from school and at the school canteen.

For each participant, we calculated the number of serves for each of the following ten food categories: (1) cereals (bread, pasta and rice); (2) vegetables and legumes (all varieties excluding tubers); (3) fruit (all varieties including dried); (4) dairy (milk, yoghurt and cheese); (5) fats/oils (butter); (6) red meat, pork, fish, poultry and eggs; (7) water; (8) SSBs; (9) extra foods high in salt or sugar or saturated fat (french fries, salty processed meats, chocolate and confectionary, cakes, pastries and biscuits); and (10) other (tubers such as cassava, yams, taro, sweet potato; noodle soup; take-away food and breakfast cereals). To gain a global understanding of how well the food consumption in rural and urban areas met the Pacific guidelines, the above ten food categories were condensed to the three main food groups for the Pacific communities, plus limited foods, limited beverages and water [19] as described in Table 1. These groups are: (1) energy foods (cereals and tubers), which should comprise 50% of the food intake corresponding to a minimum of 6 serves per day; (2) protective foods (vegetables, fruits), which should comprise 35% of food intake corresponding to a minimum of 5 serves per day; (3) bodybuilding foods (red meat, pork, fish, poultry and eggs, dairy and legumes), which should comprise 15% of all foods corresponding to a minimum of 1.5 serves per day; (4) limited foods (extra foods high in salt or sugar or saturated fat); (5) limited beverages (SSBs); and (6) water. It should be noted that extra foods and other foods like noodle soup with Maggi sauce, cakes and confectionary, as well as SSBs, are not recommended, but the number of serves of these was calculated.

#### 2.2.4. Sleep

The sleep duration was determined with the following four questions: ‘What time do you fall asleep on school days?’, ‘What time do you fall asleep on the weekend?’, ‘What time do you wake up in the school week?’ and ‘What time do you wake up on the weekend?’ There were 13 available categories for the time an adolescent might fall asleep from ‘Around 9 pm or before’ to ‘Around 3 am or later’ with a 30-min interval between each category. There were 15 available categories for the wake-up time from ‘Around 5 am or before’ to ‘Around midday or later’ with a 30-min interval between each category. Answers were converted to numerical values by using the median value of the time interval in the categorised answer or by using 30 min before (respectively after) for the first (respectively the last) category. The final sleep duration was the difference between the wake-up time and the falling-asleep time.

First, answers about sleeping duration during the school week and the weekend were separately processed and then both factors were combined to get a total sleeping duration for the full week as follows: Sleep (Total Week)=5×Sleep (Week days)+2×Sleep (weekend). Sleep durations were determined according to the recommendations from Hirshkowitz et al. [23] about sleeping, with a threshold of 9 h 30 min for these 11- to 16-year-old adolescents.

### 2.3. Statistics

Analyses were conducted using R 3.5.1. [30], with an accepted type I error probability set at α=0.05. We tested the differences between adolescents living in rural and urban areas for each parameter. For categorical parameters, the χ^2^ test was performed when Cochran’s rule was verified, otherwise Fisher’s exact test was used. For numerical parameters, Student’s *t*-test of means equality was used when the assumption of variance equality was not rejected after an F test to compare variances, otherwise the Welch test of means equality was used. The sample size (both in rural and urban Melanesian adolescents) authorised these two parametric tests. 

The percentages of adolescents meeting the dietary guidelines for each of the Pacific food groups (energy: 6 serves/day, protective: 5 serves/day, bodybuilding: 1.5 serves/day) was calculated in the whole sample and according to sex, weight status and the living area. The differences in proportions between rural and urban adolescents meeting the guidelines were tested with the χ^2^ test when Cochran’s rule was verified and otherwise with the Fisher’s exact test. 

## 3. Results 

### 3.1. SES, Anthropometry and Sleep Duration

The descriptive data, both overall and by sex, are presented in Table 2. The sample of 204 boys and 212 girls was all within the age range of 11 to 16 years. The breakdown of SES was: 11.8% high status, 11.3% intermediate status and 76.2% low status.

The percentage of overweight or obesity was 38.1% for rural and 31.7% for urban adolescents. No significant differences emerged between adolescents living in rural and urban areas, both in girls and boys. No other significant differences in demographic characteristics, such as place of residence or SES, were found.

Sleep duration in the school week or the weekend did not differ between rural and urban groups. However, average sleep duration was substantially below the international recommendations (9.50 h per night) [23] in both living areas, with 8.34 and 8.55 h per night in rural and urban areas.

### 3.2. Food Consumption and Frequency on School Days 

**Figure 1:**
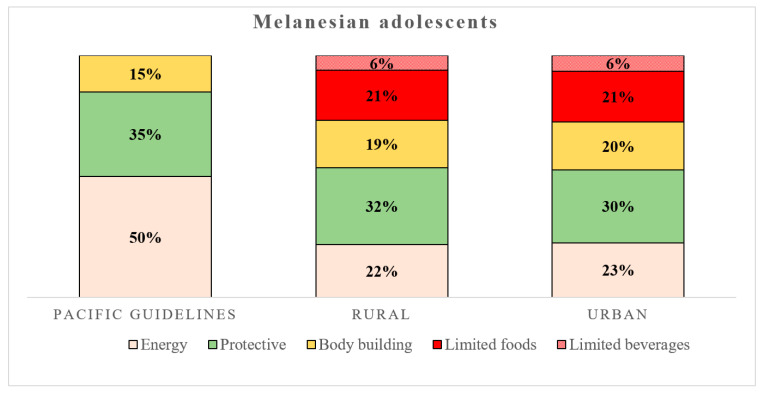
Food group proportions (percentages) and structure (yellow for energy group; green for protective group, orange for bodybuilding group, red for limited food and red and white dots for limited drinks) of rural (*n* = 312, middle column) and urban (*n* = 104, right column) adolescents compared with the Pacific guidelines (left column) [19]. Data are expressed in percentages (%) per day.

Food consumption for the energy, protective and bodybuilding groups did not significantly differ between the rural and urban adolescents (Figure 1). The extra and other foods defined as limited and SSBs showed no differences, with high consumption observed for those living in both rural and urban areas (Table 3 and Figure 1). The average contribution of the food groups for the rural and urban adolescents was, respectively: energy: 22% and 23%, protective: 32% and 30% and bodybuilding: 19% and 20% (Figure 1). Moreover, the percentage of limited food averaged 21% and limited drinks reached 6% for both rural and urban Melanesian adolescents. We also assessed the percentage of the sample meeting the Pacific guidelines for the three food groups (using number of serves compared with recommended daily intake) and found no differences between rural and urban adolescents for the whole sample, the underweight and normal-weight subgroup, or the overweight and obese subgroup (Table 3). Most adolescents met the Pacific guidelines for bodybuilding foods. 61.5% of rural and 69.2% of urban adolescents consumed sufficiently protective foods including fruits and vegetables. The recommended intake for the energy group was only achieved by 18.0% of rural and 26.9% of urban adolescents. Less than 10% of these adolescents avoided limited foods and those in urban areas who were normal or underweight all consumed these foods, with none totally avoiding them. More than half the adolescents managed to avoid SSBs.

## 4. Discussion

By focusing on food consumption and sleep, this study confirmed an advanced transition in one of the PICTs, New Caledonia. Both rural and urban Melanesian adolescents failed to meet recommendations for the consumption of traditional energy sources and instead showed high consumption of processed foods, although about three out of five avoided SSBs. Their sleep duration was low, irrespective of the place of living. Overall, these behaviour patterns may have contributed to the high rate of overweight and obesity in both rural and urban areas. 

The Melanesian adolescents had retained some of the positive aspects of the traditional diet, with 61.5% of the rural and 69.2% of the urban adolescents meeting the guidelines for the protective food group with adequate daily serves (5.55 and 5.60 serves per day in rural and urban areas, respectively). These findings contrast with the findings in Western countries like Australia, where many fail to meet the national guidelines for fruit and vegetables (albeit 7–7.5 serves is recommended in Australia) [31], and even in Fiji, where 60% of Melanesian adolescents fail to meet them [26]. In both rural and urban areas, the adolescents more than met the daily serves for the bodybuilding group, which provides the main sources of dietary protein and many micronutrients. However, rather than local traditional sources of carbohydrate-rich foods, they tended to select snack foods and two out of five drank SSBs whatever the living area. Moreover, we noted that water consumption is slightly lower in the rural areas (3.14 serves/day) when compared with the urban areas (3.39 serves/day). We previously reported on the high intake of SSBs and suggested such explanations as safety concerns about tap water and the extensive marketing of these beverages [20]. However, the amount of limited beverages consumed in the rural areas is also slightly lower on average (1.16 serves/day) when compared with the urban areas (1.36 serves/day). The differences between the rural and urban areas in beverages consumption (water and limited beverages) are not statistically significant but the urban adolescents seem to drink more beverages than their rural counterparts, especially in girls (rural: 4.10 serves/day and urban: 4.51 serves/day). Studies on other Pacific islands have shown how changes in food and beverage intakes have led to unbalanced diets and predisposed to malnutrition characterised by overweight and obesity, with possible micronutrient deficiency [32]. Nevertheless, our findings for the Melanesian adolescents of New Caledonia are described for the first time. Substitution of traditional food energy sources with highly processed foods high in sugar, fat and salt are consistent with Western diets consumed in countries where obesity is epidemic. These dietary changes might explain the high percentage of overweight and obesity (38.1% for rural and 31.7% for urban adolescents) observed in this study and those of other studies [10,11,12]. 

The pattern of lower consumption of ‘healthy food’ and higher consumption of ‘limited food’ of the Melanesians in the Pacific was apparent in both rural and urban dwellers. No differences were found for most food categories based on location. This might be because the adolescents have lunchtime meals prepared at school (part-time boarders) and some also have dinner at school (full-time boarders). These meals have standardised food intakes across regions. Other students had easy access to shops to purchase food on their way to and from school. The types of foods on offer are typically those high in added sugar, saturated fat or salt that should be limited in diets, and yet almost three in five Melanesian adolescents reported buying food in the morning journey and a little over half on the trip home. Not only are limited foods easily accessible, but they are also extensively marketed, persuading adolescents to purchase them despite their low dietary quality. The abandonment of recommended food groups in favour of ‘extra’ foods that should be limited has long been recognised in neighbouring countries like Australia, where as much as 40% of the energy in adolescent diets comes from these foods [33]. Such food patterns may result in excessive energy intake (in the present case, corresponding to 27% of daily food intake, Figure 1), which would lead to weight gain in children [34]. By replacing more nutritious foods, ‘extra’ foods might also lead to marginal intakes of some micronutrients [35,36]. 

One explanation for the current pattern of dietary intake is the reduced place of family farming in the community. Family farming has played a central role in the Melanesian community and has fed populations for decades in both rural and urban areas (urban gardens) [15,37]. Yet, population growth and climate change together have weaken food safety (Sustainable Goal Development number 2) and health (Sustainable Goal Development number 3) in the Pacific population [3]. Second, traditional foods with higher-fibre content are now juxtaposed with modern highly processed foods and beverages that are highly visible in the marketplace. Indeed, the socioeconomic transition in the Pacific region has accelerated over the past few decades and is characterised by the integration of commercial and processed foods into the traditional diet, with both contributing to food over-abundance for meals [38]. Third, both what time and how frequently meals or snacks are consumed need to be considered. One review suggested that how many and when meals are consumed throughout the day are not as important as how energy is distributed across the meals [39]. This suggests that the combination of breakfast skipping [21] and the timing and frequency of meals and snacks might play a major role in adolescent weight status. 

As one of the lifestyle components, sleep duration during the school week and the weekend was substantially below the international recommendations [23] (Table 2). Indeed, adolescents in both rural and urban areas wake up very early, as school begins between 7 and 7.30 am. When the school transport time is factored in, sleep duration is de facto reduced, with wake-up times between 5 and 5.30 am—even before 5 am for some of these families. The rhythms observed during a typical school day added to a contemporary lifestyle at home in the evening (media, screen time, etc.,) may be additional influences on food consumption, as already observed in Vanuatu adults [40]. Moreover, media messages are known to influence eating behaviours in adolescents [41] and may lead to eating disorders [42]. Childhood obesity has traditionally been ascribed to habits of high-calorie eating and sedentary lifestyles. Importantly, more recent research suggests that sleep duration may also have a role in the development of obesity, as sleep is crucially implicated in hormonal release, metabolic changes and lifestyle, all factors that contribute to overweight and obesity [43]. The exact mechanisms underlying the relationship between sleeping and overweight and obesity require further elucidation [44], but the link between insufficient sleep and weight gain through high caloric intake might involve increased ghrelin levels and decreased leptin levels, both of which stimulate appetite and the intake of excessive food [45]. In addition, it has been shown that insufficient sleep can affect food choices, resulting in lower protective food consumption and higher consumption of limited food and drinks [46]. In adolescents with sedentary activities (media use), there are many more opportunities to eat highly processed food and drinks. Not least, insufficient sleep impacts energy expenditure, with sleep-deprived people feeling sleepy and tired in the daytime, prompting them to choose sedentary activities over physical activity and exercise [4].

In the present context, the combined effects of unhealthy food behaviours, including increased consumption of limited foods and daily snacking, and reduced sleeping time most likely contribute to the high proportion of overweight and obesity in Melanesian adolescents across places of living. It is clear that these behaviours will contribute to the development of chronic diseases among the population over the long term. 

### Limitations and Strengths of the Study 

As this study was cross-sectional, we cannot point to causal relationships or long-term trends. However, we collected data directly in the participating schools: anthropometric measurements were made by trained staff during medical examinations, which ensured reliable assessments, and the FFQ was completed on days when the researchers were present. Yet, as with all self-report dietary assessments, bias may have been introduced by the participants due to recall difficulties and social desirability in the reporting.

The short FFQ presents limitations regarding the interpretation of food intake. While it was possible to group the food categories into the three recommended food groups, plus limited foods and drinks and water, the questions were not exhaustive and may not have fully captured the diversity of dietary intakes in a population undergoing nutritional transition. We did not quantitatively assess the macronutrient and micronutrient intakes or the portion sizes for the serves. Future studies will therefore include other more comprehensive dietary assessment methods and further qualitative assessment of food habits to permit a more comprehensive and powerful analysis of food behaviour in Melanesian adolescents. In addition, the use of self-reported information for sleep time duration does not inform the quality of sleep or the time of falling asleep, which might influence the global sleep of these adolescents. Another important point is energy expenditure via objective measure of accelerometery, which is known to have major impact on anthropometric parameters and could help to better understanding of adolescents’ lifestyle. So, future directions needs to consider the place of physical activity.

## 5. Conclusions

In both rural and urban areas, processed food is omnipresent in the diets of Melanesian adolescents, although some of the traditional food patterns are nevertheless still present. Overall, sleep durations are low whatever the place of living. These lifestyle factors may contribute to overweight and obesity, which lead to chronic diseases and will thus have a major impact on the Melanesian population in the coming decades. A more comprehensive approach to macro- and micronutrient intakes, combined with the assessment of physical activity levels and other lifestyle and sociodemographic factors, is needed. The findings could be used to enhance health education programs in the schools and for families in New Caledonia and other Pacific communities and perhaps for policy to maintain the healthier traditional food supply. 

## Figures and Tables

**Table 1:** Dietary intake extracted from Gwynn’s FFQ [29] was analysed using the Pacific Food Group Guidelines from the South Pacific Community [19].

Pacific Guidelines Food Groups	Main Nutrients Provided	Food	Question Extracted from Gwynn’s FFQ
Energy	Carbohydrates Vitamins Dietary fibre	Bread	How often do you eat bread (piece)? This includes baguette bread, baby bread, coconut bread, sandwich bread, etc.
		Pasta and rice	How often do you eat pasta or rice?
		Tubers	How often do you eat tubers (cassava, yam, taro, sweet potato, etc.)?
Protective	Vitamins Minerals Dietary fibre Phytochemicals Antioxidant	Vegetables	How often do you usually eat vegetables per day (for example, salad, green beans, cabbage, carrots, tomatoes, etc.)? This includes all fresh, frozen and canned vegetables.
		Fruits	How often do you eat fruits per day (for example, papaya, banana, mango, orange, apple, etc.)? This includes all fresh, dried, frozen and canned fruits.
Bodybuilding	Proteins and essential amino acids Vitamins Minerals Fatty acids Fibre (from dried beans and nuts)	Lentils, beans	How often do you eat lentils, split peas or dried beans?
		Milk	What is the total amount of milk you generally drink each day? Take into account all types of milk (brick, powder, milk consumed with cereals, etc.)
		Cheese	How often do you eat cheese?
		Yoghurt	How often do you eat yoghurt?
		Red meat	How often do you eat red meat (such as beef, deer or lamb)? This includes all steaks, ribs, roasts, minced meat, stirfries and stews.
		White meat	How often do you eat white meat like chicken?
		Fish	How often do you eat fish?
		Pork	How often do you eat pork?
		Eggs	How often do you eat eggs?
Limited beverage		SSB	How many sweetened drinks do you usually drink (juice, soda, lemonade)?
Limited food		Butter	How often do you eat your bread with butter or margarine (for example, Meadowlea)?
		Canned meat	How often do you eat canned meat (corned beef, ouaco beef, etc.)?
		Deli meats	How often do you eat cold cuts, sausages, pâté, canned ham?
		French fries	How often do you eat french fries?
		Salty snacks	How often do you eat potato chips or other salty snacks (Twisties, Doritos, etc.)?
		Sweeties	How often do you eat confectionery (lollipops, chocolate etc.)?
		Sweet foods	How often do you eat sweet foods such as sweet biscuits, cake or pastries?
		Breakfast cereals	How often do you eat breakfast cereals?
		Noodle soup	How often do you usually eat noodle soup (bowl of soup, Maggi soup, Yum Yum soup, etc.)?
		Take-away food	How often do you eat meals such as hamburgers, pizzas, fries from places selling take-away food?
Water		Water	How much water do you usually drink each day? It can be tap water or bottled water (a small bottle = two glasses).

**Table 2:** Anthropometric (weight, height, weight status) and sociodemographic characteristics (SES) and sleep duration according to the adolescents’ living area (rural or urban) and sex. Numbers represent ‘Mean (Standard deviation)’ for the numerical variables (Age, Anthropometry and Sleep duration) and ‘Size (%)’ for the categorical variables (SES, Weight status and Meals). Statistical significance was noted in the *p*-value column.

		Whole Sample			Female			Male		
		Rural (*n* = 312)	Urban (*n* = 104)	*p*-Value	Rural (*n* = 167)	Urban (*n* = 45)	*p*-Value	Rural (*n* = 145)	Urban (*n* = 59)	*p*-Value
Subjects [Mean (sd)]	Age (months)	160.52 (15.21)	156.63 (12.60)	0.011	162.16 (14.22)	156.73 (11.68)	0.020	158.64 (16.11)	156.56 (13.36)	0.381
SES [*n* (%)]	Higher	35 (11.2%)	14 (13.4%)	0.060	17 (10.2%)	4 (8.9%)	0.401	18 (12.4%)	10 (17.0%)	0.110
	Intermediate	29 (9.3%)	18 (17.3%)		17 (10.2%)	8 (17.8%)		12 (8.3%)	10 (17.0%)	
	Lower	245 (78.5%)	72 (69.2%)		131 (78.4%)	33 (73.3%)		114 (78.6%)	39 (66.1%)	
Anthropometry [Mean (sd)]	Height (cm)	156.5 (8.9)	157.3 (8.9)	0.417	156.7 (7.2)	157.0 (5.3)	0.762	156.2 (10.5)	157.5 (11.0)	0.427
	Weight (kg)	54.4 (14.4)	54.1 (14.0)	0.846	55.4 (13.2)	55.4 (11.6)	1.000	53.3 (15.7)	53.1 (15.6)	0.942
Weight status [*n* (%)]	Underweight and Normal	193 (61.9%)	71 (68.3%)	0.290	98 (58.7%)	29 (64.4%)	0.597	95 (65.5%)	42 (71.2%)	0.537
	Overweight and obese	119 (38.1%)	33 (31.7%)		69 (41.3%)	16 (35.6%)		50 (34.5%)	17 (28.8%)	
Sleep duration [Mean (sd)]	Weekday (h/day)	8.16 (1.10)	8.31 (1.29)	0.302	8.15 (1.11)	8.29 (1.26)	0.459	8.18 (1.09)	8.32 (1.32)	0.427
	Weekend (h/day)	8.80 (1.69)	8.84 (1.99)	0.854	8.96 (1.63)	9.01 (2.02)	0.854	8.61 (1.74)	8.70 (1.98)	0.740
	All week (h/week)	58.40 (7.03)	59.21 (8.46)	0.380	58.65 (7.22)	59.47 (8.35)	0.516	58.12 (6.81)	59.02 (8.61)	0.475
Meals [*n* (%)]	Lunch at school	205 (65.7%)	96 (92.3%)	<0.001	108 (64.7%)	43 (95.6%)	<0.001	97 (66.9%)	53 (89.8%)	<0.001
	No lunch at school	15 (4.8%)	8 (7.7%)		6 (3.6%)	2 (4.4%)		9 (6.2%)	6 (10.2%)	
	In boarding school	92 (29.5%)	0 (0.0%)		53 (31.7%)	0 (0.0%)		39 (26.9%)	0 (0.0%)	

**Table 3:** Food frequency, food group consumption expressed in serves per week (with school meals according to each adolescent‘s living area: rural or urban) and sex. Statistical significance was noted in the *p*-value column.

		Whole Sample					Female					Male				
		Serves per Day [Mean (sd)]		% Meeting the Guidelines			Serves per Day [Mean (sd)]		% Meeting the Guidelines			Serves per Day [Mean (sd)]		% Meeting the Guidelines		
		Rural	Urban	Rural	Urban	*p*-Values	Rural	Urban	Rural	Urban	*p*-Values	Rural	Urban	Rural	Urban	*p*-Values
Whole sample	Energy group	4.06 (2.20)	4.56 (2.54)	18.0	26.9	0.067	4.04 (2.28)	4.08 (2.30)	19.2	20.0	1.000	4.07 (2.12)	4.92 (2.66)	16.6	32.2	0.022
	Protective group	5.55 (2.30)	5.60 (2.06)	61.5	69.2	0.196	5.47 (2.28)	5.67 (2.09)	60.5	73.3	0.158	5.65 (2.33)	5.55 (2.05)	62.8	66.1	0.772
	Bodybuilding group	3.51 (1.77)	3.87 (2.15)	88.5	94.2	0.133	3.27 (1.65)	3.40 (1.83)	86.2	91.1	0.535	3.79 (1.85)	4.23 (2.32)	91.0	96.6	0.277
	Limited foods	4.01 (2.63)	4.41 (2.80)	6.4	1.9	0.129	4.07 (2.63)	4.20 (2.82)	4.8	4.4	1.000	3.95 (2.64)	4.58 (2.80)	8.3	0.0	0.020
	Limited beverages	1.16 (1.22)	1.36 (1.37)	61.5	56.7	0.452	1.11 (1.19)	1.40 (1.37)	62.9	53.3	0.321	1.21 (1.25)	1.33 (1.37)	60.0	59.3	1.000
	Water	3.14 (1.14)	3.39 (1.07)				2.99 (1.19)	3.11 (1.06)				3.30 (1.07)	3.61 (1.03)			
Underweight and normal weight	Energy group	4.10 (2.23)	4.71 (2.50)	18.1	28.1	0.108	3.88 (2.26)	4.09 (2.13)	16.3	17.2	1.000	4.32 (2.19)	5.14 (2.66)	20.0	35.7	0.080
	Protective group	5.54 (2.36)	5.70 (2.12)	60.1	69.0	0.237	5.23 (2.31)	5.74 (2.24)	55.1	72.4	0.147	5.86 (2.39)	5.67 (2.06)	65.3	66.7	1.000
	Bodybuilding group	3.53 (1.76)	3.81 (2.14)	89.1	94.4	0.292	3.25 (1.65)	3.07 (1.61)	85.7	89.7	0.813	3.83 (1.83)	4.32 (2.33)	92.6	97.6	0.452
	Limited foods	4.32 (2.81)	4.55 (2.91)	6.2	0.0	0.041	4.26 (2.82)	4.21 (2.85)	6.1	0.0	0.335	4.38 (2.80)	4.78 (2.97)	6.3	0.0	0.177
	Limited beverages	1.21 (1.29)	1.40 (1.35)	61.7	53.5	0.293	1.19 (1.25)	1.37 (1.37)	60.2	89.7	0.788	1.23 (1.34)	1.43 (1.36)	63.1	52.4	0.319
	Water	3.09 (1.15)	3.39 (1.10)				2.91 (1.20)	3.17 (1.11)				3.27 (1.08)	3.54 (1.08)			
Overweight and obese	Energy group	3.99 (2.17)	4.23 (2.63)	17.7	24.2	0.547	4.28 (2.32)	4.07 (2.66)	23.3	25.0	1.000	3.60 (1.90)	4.37 (2.66)	10.0	23.5	0.216
	Protective group	5.58 (2.20)	5.40 (1.93)	63.9	69.7	0.678	5.81 (2.20)	5.54 (1.84)	68.1	75.0	0.812	5.25 (2.17)	5.26 (2.06)	58.0	64.7	0.841
	Bodybuilding group	3.47 (1.77)	4.00 (2.20)	87.4	93.9	0.457	3.29 (1.67)	3.99 (2.10)	87.0	93.8	0.742	3.72 (1.90)	4.01 (2.36)	88.0	94.1	0.669
	Limited foods	3.52 (2.25)	4.12 (2.55)	6.7	6.1	1.000	3.80 (2.34)	4.17 (2.84)	2.9	12.5	0.328	3.13 (2.08)	4.08 (2.34)	12.0	0.0	0.325
	Limited beverages	1.07 (1.09)	1.27 (1.40)	61.3	63.6	0.970	0.99 (1.10)	1.45 (1.41)	66.7	50.0	0.337	1.18 (1.08)	1.11 (1.42)	54.0	76.5	0.179
	Water	3.21 (1.13)	3.39 (1.02)				3.11 (1.17)	3.00 (0.99)				3.36 (1.07)	3.76 (0.92)			
